# Impact and Value of Hospital Antibiotic Stewardship: Retrospective Pre-COVID-19-Pandemic Analysis

**DOI:** 10.3390/jcm11154412

**Published:** 2022-07-29

**Authors:** Maria Costantino, Valeria Conti, Graziamaria Corbi, Alessandra Anna Iannelli, Francesco Marongiu, Martina Torsiello, Antonio Della Vecchia, Carmine Sellitto, Armando Genovese, Giuseppina Moccia, Amelia Filippelli, Francesco De Caro

**Affiliations:** 1 Department of Medicine, Surgery and Dentistry “Scuola Medica Salernitana”, 84081 Baronissi, Italy; vconti@unisa.it (V.C.); mtorsiello@unisa.it (M.T.); csellitto@unisa.it (C.S.); gmoccia@unisa.it (G.M.); afilippelli@unisa.it (A.F.); fdecaro@unisa.it (F.D.C.); 2 University Hospital “San Giovanni di Dio e Ruggi d’Aragona”, 84121 Salerno, Italy; alessandra.iannelli@sangiovannieruggi.it (A.A.I.); armandogeno@gmail.com (A.G.); 3 Association Non-Profit F.I.R.S.Thermae (Interdisciplinary Training, Researches and Spa Sciences), 80078 Naples, Italy; fmarongiu@unisa.it; 4 Department of Translational Medical Sciences, University of Naples Federico II, 80131 Naples, Italy; graziamaria.corbi@unina.it; 5 Italian Society of Gerontology and Geriatrics (SIGG), 50129 Florence, Italy; 6 Department of Industrial Engineering, University of Salerno, 84084 Fisciano, Italy; 7 General Directorate of Health Planning, Office 3—Italian Ministry of Health, 00144 Roma, Italy; a.dellavecchia@sanita.it

**Keywords:** Antimicrobial stewardship, antibiotic resistance, COVID-19

## Abstract

Aim: “Antimicrobial stewardship” (AMS) is defined as a healthcare-system-wide approach to promoting and monitoring the judicious use of antimicrobials to preserve their future effectiveness. Therefore, we structured an observational study to monitor the hospital trend of antibiotic consumption and related expenditure before the COVID-19 pandemic and to evaluate how much AMS could affect this trend. Methods: The research covered the antibiotic prescriptions at the University Hospital (U.H.) “San Giovanni di Dio e Ruggi d’Aragona”, Salerno, Italy, comparing data on the therapies prescribed from 1 January to 31 December 2017 (27,384 patients) with those collected during the same period in 2019 (27,047 patients). Results: Unlike national data, our results highlighted a decreasing trend in the consumption of antibiotics that did not concern only carbapenems and fluoroquinolones, but also the third-generation cephalosporins. Noteworthily, there was also a reduction in 2019 compared with 2017 in the consumption of colistin, an antibiotic towards which an increase in bacterial resistance in animals has been found nationally. In agreement with the national data, our research confirms a trend of an increase (+3.7%) in the total antibiotic consumption corresponding to more than 26% and 29% reductions in the total and therapy per-day costs, respectively. Conclusions: The results show a positive impact of the AMS at the University Hospital “San Giovanni di Dio e Ruggi d’Aragona”.

## 1. Introduction

“Antimicrobial stewardship” (AMS) defines a healthcare-system-wide approach to promoting and monitoring the judicious use of antimicrobials to preserve their future effectiveness [1,2,3,4].

In hospital, the term indicates the monitoring of an antimicrobial’s use through a standardized evidence-based approach, to reduce the selection and spread of resistant germs and the adverse effects related to the use of antibiotics, and ultimately contain the costs [5].

Currently, antibiotic resistance has become a worldwide problem with serious economic, clinical and public health implications, and most deaths are caused by resistance to antibiotics used in lower respiratory tract infections, such as pneumonia, and blood and intra-abdominal infections [6,7,8,9].

In Europe, Italy has the highest incidence of antibiotic resistance to the main classes of antibiotics used in hospitals [9,10]. In fact, antibiotic-resistant infections typically contracted in hospitals or other health facilities can prolong a patient’s length of stay and are often associated with the need for further treatments, increasing the risk of drug–drug interactions and adverse events [11]. Therefore, there is an urgent need to set up strategies to promote better use of antibiotics [12,13,14,15] and create a comprehensive interdisciplinary network to share data at local and national levels, as well as among different countries [16].

In 2014, a cost of approximately EUR 320 million was attributed to antimicrobial resistance (AMR) in Italy, and AMR costs are projected to reach EUR 2 billion by 2050 in the absence of specific interventions [17].

According to the Eurobarometer, Italians are the least aware of this enormous social and healthcare burden and they often ignore the fact that the overuse of the available antibiotics can render them ineffective [18]. A 2019 survey [19] found that only half of the national sample considered claimed to know what antibiotic resistance was, while 21.6% had heard of it, and 28.3% (mostly the elderly) admitted to not knowing what AMR was.

The hospital setting represents an area where specific interventions are urgently needed. Interventions to limit the inappropriate use of antibiotics and healthcare-associated infections (HAIs) include drug-consumption monitoring, vaccine use and the improvement of healthcare professionals’ awareness in these fields [20,21,22].

Therefore, it is necessary to screen the hospital consumption of antibiotics to counteract antibiotic resistance, reduce adverse reactions related to their use and limit the lengths of stay.

Based on these considerations, we performed an observational study to monitor the hospital trend of antibiotic consumption and related expenditure, and to evaluate the impact of the AMS established at the University Hospital “San Giovanni di Dio e Ruggi d’Aragona (U.H. Ruggi).

## 2. Materials and Methods

### 2.1. Intervention

The research covered the antibiotic prescriptions at U.H. Ruggi, Salerno, Italy.

Data on the antibiotics prescribed from 1 January to 31 December 2017 (*n* = 27,384 patients) were compared with those collected during the same period in 2019 (*n* = 27,047).

The year 2019 was considered because it corresponds to the period immediately preceding the outbreak of the COVID-19 pandemic. The year 2017 was considered because corresponds to the year before the implementation of AMS at the U.H. Ruggi. 

In particular, from the beginning of 2018 until the end of 2019, in the U.H. Ruggi, the AMS was implemented. 

The interventions implemented as part of the AMS firstly included the establishment of a Committee for the Control of Hospital Infections (C.I.O.) and the purchase of equipment for sanitizing environments, such as ozonators, which were very useful even during the COVID-19 pandemic that broke out in early 2020. Prospective audits on antibiotic use were conducted 2 times a month, encouraging direct interaction and feedback among the prescribers. The audits foresaw the evaluation of the routine clinical practice, literature revision and EMA/AIFA note acquisition, leading to an update and modification of the in-hospital guidelines where appropriate.

Regular meetings were held, and monographs focusing on the management of resistant bacteria, especially those resistant to third-generation cephalosporins, fluoroquinolones and carbapenems, were published.

A specific control measure was the introduction of a justified request form for antibiotic prescriptions, considered an essential component in the management of AMR [9,23]. 

Other interventions included receiving updated guidelines on antibiotic prophylaxis, empirical de-escalation therapy based on the results of the crop test response, and promoting a switch from intravenous to oral drug administration.

The study obtained the approval of the Ethics Committee Campania Sud-Naples, Italy (N. 0098507—May 2021).

### 2.2. Outcomes

In 2019 and 2017, the following outcome parameters were analyzed: the types of consumed antibiotics and related expenditure; diagnosis-related group (DRG) weight means, which are numbers that reflect relative resource consumption as measured by the relative hospital’s charges [24,25] and relate pharmaceutical consumption to the intensity of the services provided; the consumption of some therapeutic categories of antibiotics (i.e., fluoroquinolones, carbapenems, cephalosporins, macrolides, polymyxins and lincosamides) that are important for the Italian National Plan to Combat Antibiotic Resistance (hereinafter referred to as 2017–2020 PNCAR) [12,15,26], in hospital wards that are considered strategic for the fight against AMR (Infectious Diseases, Cardiac Surgery, Orthopaedics and Traumatology, Resuscitation, General Medicine, Pneumology, Obstetrics and Gynecology, Urology, Intensive Cardiac Care Unit, Emergency Surgery, General Surgery and Emergency Medicine); the consumption of antibiotics in alignment with the World Health Organization (WHO) Access, Watch, Reserve (AWaRe) classification [27,28].

The antibiotics were classified using the Anatomical Therapeutic Chemical Classification System (ATC) [29]. Consumption data were obtained from the information flows provided by the hospital pharmacy as the number of antibiotic packs distributed each month to every single ward of the U.H. Ruggi and expressed as the defined daily dose (DDD), a statistical measure of drug consumption, defined by the WHO Collaborating Centre for Drug Statistics Methodology, and used in combination with the ATC Code drug classification system for grouping related drugs.

### 2.3. Statistical Analysis

The analysis of the data obtained from the current information flows was carried out using the MariaDB database, queried using Structured Query Language (SQL), a standardized database language based on a relational model.

The MariaDB is an open-source “Relational Database Management System” (MDBMS) based on a relational model, i.e., based on tables and relationships and compatible drop-in replacements for the widely used MySQL database technology.

## 3. Results

The U.H. Ruggi includes 74 operating units with 850 beds in total. Table 1 shows the main information on the antibiotic consumption in the two study periods examined. 

The comparative analysis of the 2019 data versus those collected in 2017 showed an overall increase of over 3% in the annual DDD consumption of antibiotics, with an increase of over 4% in DDD/100 bed-days, while reductions higher than 26% and 29% in the total and average costs of therapy per day were observed, respectively (Table 1). The increase in antibiotic consumption could be explained by a slight raise in patients’ complexity as showed by the DRG weight mean in 2019.

**Figure 1:**
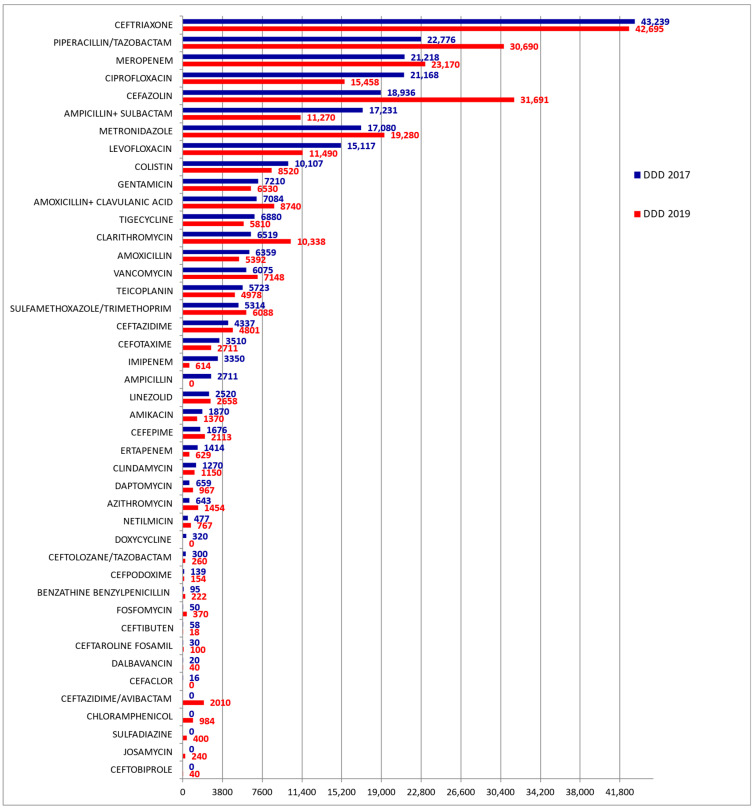
Types of antibiotics with relative DDD consumed at the U.H. during 2017 and 2019.

The data analysis shows that ceftriaxone was the most used antibiotic during the two years considered, although a slight decrease in its consumption was observed in 2019 versus 2017 (Figure 1). 

Moreover, among the classes of antibacterial agents strategic for the 2017–2020 PNCAR, a significant (*p* < 0.05) reduction of more than 25% was observed in the consumption of fluoroquinolones (DDD) in 2019 compared to 2017 (Table 2). This reduction concerns both ciprofloxacin and levofloxacin (Figure 1).

Regarding cephalosporins, also considered important for the 2017–2020 PNCAR, a more than 19% increase in DDD (*p* < 0.05) was observed in 2019 vs. 2017 (Table 2), which mainly involved the use of first- and fourth-/fifth-generation molecules (Figure 1).

Table 3 shows the antibiotic types and DDD consumed during the years 2019 and 2017 according to the WHO AWaRe classification. The data highlight that, during 2019, the percentual consumption of antibiotics categorized as Access increased, while that of the antibiotics listed in the Watch and Reserve category decreased (Table 3 and Table S1). There was also a large increase in the cefazoline DDD consumed in 2019 versus 2017 (Figure 1).

Moreover, by considering the percentual variation (Δ%) of the total DDD consumed in alignment with the WHO AWaRe classification, a statistically significantly increased (+8.9%) consumption of the Access and Watch (+1.5%) group of antibiotics (both *p* < 0.05) and a reduction in the Reserve one (−0.5%, *p* < 0.05) in 2019 versus 2017 were found (Table 3).

Altogether, these findings, also connected with a slight increase in the DRG mean weight, demonstrate that more attention is being paid to the use of the antibiotics as a result of the AMS’ introduction in 2019, and it could also partially explain the optimization of the cost experienced in 2019 compared to 2017 (Table 1).

The analysis (Tables S2–S5) of the consumption of the antibiotic classes important for PNCAR in hospital wards considered strategic for the AMR in 2019 versus 2017 highlighted a reduced consumption of fluoroquinolones in all the wards except the urology ones, where an increase was observed only for ciprofloxacin (Table S4).

Regarding carbapenems, there was a reduction in the total annual consumption of DDD in several wards (Tables S2, S4 and S5). In particular, the increased use of carbapenems in the surgery wards, especially when used in antimicrobial prophylaxis, represents one of the most common reasons for AMR. Therefore, the observed reduction in the use of these drugs in surgical wards could represent a milestone in AMR prevention (Tables S2 and S5).

For cephalosporins, the data showed an annual decrease in use in cardiac surgery, urology and emergency medicine (Tables S2, S4 and S5). We also observed a reduction in the third-generation cephalosporins in the wards of orthopedics and traumatology, pneumology, urology and emergency medicine (Tables S2–S5).

## 4. Discussion

In Italy, Campania is one of the regions with very high levels of AMR, where the percentage of the resistance to the third-generation cephalosporins, fluoroquinolones and carbapenems is higher compared to that in both the rest of Italy and Europe [30,31,32,33].

Altogether, our observational study, performed in a hospital in the Campania Region, highlights the importance of AMS, whose main aim is to promote prescriptive appropriateness for available antibiotics to improve their effectiveness and limit AMR diffusion.

The results show a positive impact of the AMS program implemented in 2018 and 2019 at the University Hospital “San Giovanni di Dio e Ruggi d’Aragona”. Interestingly, comparing 2019 to 2017 (a year in which AMS had not been implemented), reduced consumption of carbapenems, antibiotics towards which Enterobacteria have manifested an abnormal progression of resistance, was recorded. This suggests a more targeted use of this antibiotic class with microbiological support. There was also a reduction (more than 25%) in the consumption of fluoroquinolones, even though, in the Campania region, there is a consumption above the European average. It should be noted that the Italian 2017–2020 PNCAR envisaged, as a process indicator, a reduction in their consumption of at least 10% by 2020, which was largely achieved and exceeded in the University Hospital in 2019.

In our study, unlike national data, the decreasing trend in the consumption of antibiotics did not concern only carbapenems and fluoroquinolones, but also the third-generation cephalosporins, for which a reduction in consumption equal to −5.4% was found.

Noteworthily, there was also a reduction in 2019 versus 2017 in the consumption of colistin, an antibiotic towards which an increase in AMR has been found in animals. It should be noted that colistin is considered the last bulwark against bacteria.

The reduction in the annual consumption for 2019 versus 2017 of fluoroquinolones, carbapenems, colistin and lincosamides was observed in most of the hospital wards considered and reputed strategic by the National Italian PNCAR for the control of AMR. A reduction was observed in urology, cardiac surgery and emergency medicine wards for the third-generation cephalosporins and in resuscitation, obstetrics and gynecology, and cardiac surgery wards for macrolides.

The positive impact of the AMS program implemented in the U.H. Ruggi, which included prospective audits on antibiotic use, is also confirmed by data on the consumption of antibiotics classified as Access, Watch and Reserve. Indeed, we found that the percentual distribution of the antibiotics in 2019 showed a decrease in both the Watch and Reserve categories in favor of the Access one.

Concerning the antibiotics belonging to the Access category, it should be noted that, in 2019 compared to 2017, there was a large increase in the use of cefazolin, which is the drug of choice for surgical prophylaxis in monotherapy or in association with metronidazole such as in the management of intra-abdominal infections.

Moreover, when comparing the 2019 to 2017 data, an increase in the consumption (Δ value = +8.9%) of Access antibiotics that should always be used as first-line treatments for a wide range of common infections was observed, and a reduction (Δ value = −0.5%) in Reserve antibiotics (Table 3), used as the last option under strict monitoring to avoid the onset of resistance, was observed. Instead, the Watch antibiotics, recommended only for specific indications, given their high resistance-inducing potential, showed an increase in annual consumption (Δ value = +1.5%) in 2019 vs. 2017. These last findings could be explained by a slight raise in patients’ complexity as showed by the DRG weight mean in 2019 with respect to 2017. Altogether, these findings suggest that more attention is paid to the use of the antibiotics as a result of the AMS’ introduction in 2019, and it could also partially explain the optimization of the costs experienced in 2019 compared to 2017; the average cost of daily therapy was equal to 2.9 in 2019, compared with 4.1 in 2017.

The promotion of a constant dialog between healthcare professionals, the introduction of a justified nominative request form for antibiotic prescriptions, and the receipt of the updated guidelines on antibiotic prophylaxis undoubtedly helped to reduce the inappropriate use of antibiotics. The interventions adopted as part of the AMS have proved to be particularly useful to promote more rational use of fluoroquinolones, carbapenems and third-generation cephalosporins, whose effectiveness is threatened by the emergence of multidrug-resistant bacteria [34,35,36].

The major limitation of this study is that the results regard only one hospital center. However, at the same time, a careful analysis was conducted using a large amount of data. Another major limitation is the lack of information on the type and severity of the disease responsible for the antibiotic prescription, which allows only hypothesizing regarding the effect of the AMS intervention. Therefore, further studies are necessary to better clarify this issue.

## 5. Conclusions

The war against antimicrobial resistance should aim to discover and make available new antimicrobials and alternative treatments [22,37,38,39] and improve the use of existing antimicrobials. This milestone cannot be reached without analyzing real-world data.

Increasing the awareness of AMR, together with specific interventions, will allow us to better combat antibiotic resistance in the future.

## Figures and Tables

**Table 1:** Consumption, expressed in DDD and DDD/100 bed-days, of antibiotics and related costs, in EUR, in the year 2017 versus 2019.

Parameters	Time Period (Years)		% Variation
	2017	2019	
DDD of antibiotics consumed	263,501	273,360	+3.7
Costs	1,072,083	783,682	−26.9
DDD/100 bed-days	107	112	+4.7
DRG weight means	1.26	1.29	+2.4
Average cost of therapy per day	4.1	2.9	−29.3

**Table 2:** Consumption, expressed in DDD, of fluoroquinolones, carbapenems, cephalosporins, macrolides, polymyxins and lincosamides in 2017 versus 2019.

Antibiotic Category	DDD Consumed in 2017	DDD Consumed in 2019	*p*	Δ% in 2017 vs. 2019
Fluoroquinolones, n(%)	36,285 (13.8)	26,948 (9.9)	<0.05	−25.7
Carbapenems, n(%)	25,982 (9.9)	24,413 (8.9)	<0.05	−6.0
Cephalosporins, n(%)	72,241 (27.4)	86,593 (31.7)	<0.05	+19.9
Macrolides, n(%)	7162 (2.7)	12,032 (4.4)	<0.05	+68.0
Polymyxins, n(%)	10,107 (3.8)	8520 (3.1)	<0.05	−15.7
Lincosamides, n(%)	1270 (0.48)	1150 (0.42)	<0.05	−9.4

**Table 3:** Percentage of total DDD consumed in alignment with the WHO AWaRe classification, in 2017 and 2019.

AWaRe Category	DDD Consumed in 2017	DDD Consumed in 2019	*p*	Δ% in 2017 vs. 2019
ACCESS, n(%)	85,480 (32.4)	93,117 (34.1)	<0.05	+8.9
WATCH, n(%)	157,505 (59.8)	159,838 (58.5)	<0.05	+1.5
RESERVE, n(%)	20,516 (7.8)	20,405 (7.5)	<0.05	−0.5

## Data Availability

Not applicable.

## Supplementary Materials

The following supporting information can be downloaded at: https://www.mdpi.com/article/10.3390/jcm11154412/s1, Figure S1: Annual trend of the consumption of antibiotic classes, expressed in % of total DDD consumed, at the U.H. during 2017 and 2019. Table S1: Total DDD of antibiotics consumed in alignment with the W.H.O. AWaRe classification, in 2017 and 2019. Table S2: Annual consumption, expressed in % of total DDD antibiotics consumed, of fluoroquinolones, carbapenems, cephalosporins, macrolides, polymyxins, and lincosamides in some hospital wards of U.H. Ruggi in 2017 versus 2019. Table S3: Annual consumption, expressed in % of total DDD antibiotics consumed, of fluoroquinolones, carbapenems, cephalosporins, macrolides, polymyxins and lincosamides in some hospital wards of U.H. Ruggi in 2017 versus 2019. Table S4: Annual consumption, expressed in % of total DDD antibiotics consumed, of fluoroquinolones, carbapenems, cephalosporins, macrolides, polymyxins, and lincosamides in some hospital wards of U.H. Ruggi in 2017 versus 2019. Table S5: Annual consumption, expressed in % of total DDD antibiotics consumed, of fluoroquinolones, carbapenems, cephalosporins, macrolides, polymyxins, and lincosamides in some hospital wards of U.H. Ruggi in 2017 versus 2019.
