# Supplementary material for: Impact and Value of Hospital Antibiotic Stewardship: Retrospective Pre-COVID-19-Pandemic Analysis
Source: J Clin Med. 2022 Jul 29;11(15):4412. doi: 10.3390/jcm11154412 (PMC9369048; doi:10.3390/jcm11154412)
Supplement: Supplementary file 1 [file jcm-11-04412-s001.zip › jcm-1792219-supplementary.pdf]

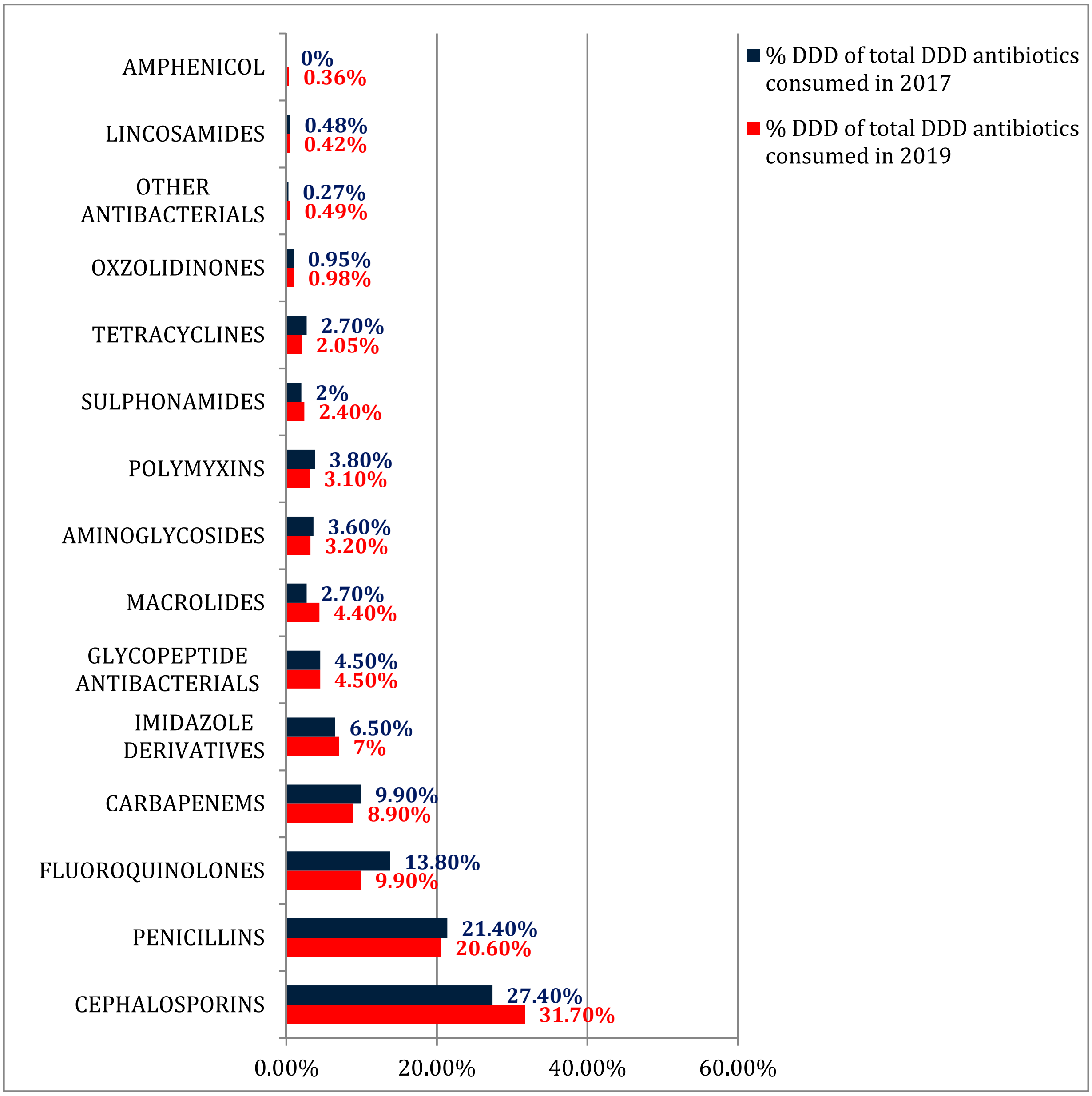

Figure S1. Annual trend of the consumption of antibiotic classes, expressed in % of total DDD consumed, at the U.H. during 2017 and 2019.

Table S1. Total DDD of antibiotics consumed in alignment with the W.H.O. AWaRe classification, in 2017 and 2019.

CATEGORY AWaRe	DDD Consumed in 2017	DDD Consumed in 2019
**ACCESS ANTIBIOTICS**		
chloramphenicol	0	984
sulfamethoxazole+trimethoprim	5314	6088
amikacin	1870	1370
metronidazole	17,080	19,280
gentamicin	7210	6530
ampicillin+sulbactam	17,231	11,270
benzathine benzylpenicillin	95	222
sulfadiazine	0	400
clindamycin	1270	1150
amoxicillin	6359	5392
ampicillin	2711	0
amoxicillin+Clavulanic acid	7084	8740
cefazolin	18,936	31,691
doxycycline	320	0
**WATCH ANTIBIOTICS**		
piperacillin/tazobactam	22,776	30,690
cefpodoxime	139	154
cephachlor	16	0
cefepime	1676	2113
ceftriaxone	43,239	42,695
ceftibuten	58	18
cefotaxime	3510	2711
ceftazidime	4337	4801
levofloxacin	15,117	11,490
ciprofloxacin	21,168	15,458
meropenem	21,218	23,170
ertapenem	1414	629
imipenem/cilastatin	3350	614
clarithromycin	6519	10,338
azithromycin	643	1454
fosfomycin	50	370
josamycin	0	240
netilmicin	477	797
teicoplanin	5723	4978
vancomycin	6075	7148
**RESERVE ANTIBIOTICS**		
colistin	10,107	8520
dalbavancin	20	40
tigecycline	6880	5810
daptomycin	659	967
linezolid	2520	2658
ceftobiprole	0	40
ceftaroline fosamil	30	100
ceftolozane+tazobactam	300	260
ceftazidime/avibactam	0	2010

Table S2. Annual consumption, expressed in % of total DDD antibiotics consumed, of fluoroquinolones, carbapenems, cephalosporins, macrolides, polymyxins, and lincosamides in some hospital wards of U.H. Ruggi in 2017 versus 2019.

HOSPITAL WARD: INFECTIOUS DISEASES	*% of total DDD antibiotics consumed 2017*	*% of total DDD antibiotics consumed 2019*	*Variation % in 2019 vs. 2017*	*HOSPITAL WARD: CARDIAC SURGERY*	*% of total DDD antibiotics consumed 2017*	*% of total DDD antibiotics consumed 2019*	*Variation % in 2019 vs. 2017*	*HOSPITAL WARD: ORTHOPAEDICS AND TRAUMATOLOGY*	*% of total DDD antibiotics consumed 2017*	*% of total DDD antibiotics consumed 2019*	*Variation % in 2019 vs. 2017*
FLUOROQUINOLONES	8.6%	3.7%	-5%	FLUOROQUINOLONES	10.3%	6.9%	-3.4%	FLUOROQUINOLONES	10.0%	5.4%	-4.6%
levofloxacin	6.1%	3.2%	-3%	*levofloxacin*	3.9%	3.7%	-0.2%	*levofloxacin*	1.8%	2.1%	+0.3%
ciprofloxacin	2.5%	0.5%	-2%	*ciprofloxacin*	6.4%	3.2%	-3.2%	*ciprofloxacin*	8.2%	3.3%	-4.9%
CARBAPENEMS	18.1%	8.9%	-9%	CARBAPENEMS	8.1%	1.9%	-6.2%	CARBAPENEMS	9.9%	4.4%	-5.5%
meropenem	16.3%	7.8%	-8.5%	*meropenem*	7.3%	1.9%	-5.4%	*meropenem*	9.5%	4.4%	-5.1%
ertapenem	0.8%	1.1%	+0.3%	*ertapenem*	0.2%	0%	-0.2%	*ertapenem*	0.1%	0%	-0.1%
imipenem/cilastatin	1.0%	0%	-1%	*imipenem/cilastatin*	0.6%	0%	-0.6%	*imipenem/cilastatin*	0.3%	0%	-0.3%
CEPHALOSPORINS	7.0%	8.9%	+1.9%	CEPHALOSPORINS	32.2%	30.8%	-1.4%	CEPHALOSPORINS	38.9%	57.7%	+18.8%
ceftobiprole	0%	0.1%	+0.1%	*cefazolin*	29.6%	25.9%	-3.7%	*cefazolin*	28.2%	52.2%	+24.0%
ceftolozane+tazobactam	0.8%	0.6%	-0.2%	*ceftazidime*	1.6%	2.8%	+1.2%	*ceftazidime*	0.6%	0.5%	-0.1%
ceftaroline fosamil	0.2%	0.3%	+0.1%	*ceftazidime+avibactam*	0%	1.3%	+1.3%	*ceftriaxone*	9.7%	4.9%	-4.8%
ceftazidime+avibactam	0%	1.6%	+1.6%	*ceftriaxone*	0.2%	0.7%	+0.5%	*cefepime*	0.4%	0.1%	-0.3%
ceftazidime	2.0%	2.0%	0%	*cefepime*	0.8%	0.1%	-0.7%				
ceftriaxone	3.7%	4.4%	+0.7%								
cefotaxime	0.3%	0%	-0.3%								
MACROLIDES	1.0%	1.8%	+0.8%	MACROLIDES	0.2%	0%	-0.2%	MACROLIDES	1.2%	1.6%	+0.4%
clarithromycin	1.0%	1.1%	+0.1%	*clarithromycin*	0.2%	0%		*clarithromycin*	1.16%	1.4%	+0.24%
azithromycin	0%	0.7%	+0.7%					*azithromycin*	0.04%	0.2%	+0.16%
POLYMYXINS	2.3%	6.0%	+ 3.7%	POLYMYXINS	3.1%	2.7%	-0.4%	POLYMYXINS	9.6%	2.1%	-7.5%
colistin				*colistin*				*colistin*			
LINCOSAMIDES	0.1%	1.8%	+1.7%	LINCOSAMIDES	0%	0%	----	LINCOSAMIDES	0.1%	0%	-0.1%
clindamycin								*clindamycin*			

Table S3. Annual consumption, expressed in % of total DDD antibiotics consumed, of fluoroquinolones, carbapenems, cephalosporins, macrolides, polymyxins and lincosamides in some hospital wards of U.H. Ruggi in 2017 versus 2019.

**HOSPITAL WARD: RESUSCITATION**	**% of total DDD antibiotics consumed 2017**	**% of total DDD antibiotics consumed 2019**	**Variation % in 2019 vs. 2017**	**HOSPITAL WARD: GENERAL MEDICINE**	**% of total DDD antibiotics consumed 2017**	**% of total DDD antibiotics consumed 2019**	**Variation % in 2019 vs. 2017**	**HOSPITAL WARD: PNEUMOLOGY**	**% of total DDD antibiotics consumed 2017**	**% of total DDD antibiotics consumed 2019**	**Variation % in 2019 vs. 2017**
FLUOROQUINOLONES	7.4%	2.6%	-4.8%	*FLUOROQUINOLONES*	18.5%	13.2%	-5.3%	*FLUOROQUINOLONES*	25.6%	19.6%	-6.0%
levofloxacin	3.8%	1.0%	-2.8%	*levofloxacin*	6.5%	5.5%	-1.0%	*levofloxacin*	10.0%	8.5%	-1.5%
ciprofloxacin	3.6%	1.6%	-2.0%	*ciprofloxacin*	12.0%	7.7%	-4.3%	*ciprofloxacin*	15.6%	11.1%	-4.5%
CARBAPENEMS	13.8%	21.0%	+7.2%	*CARBAPENEMS*	12.2%	16.3%	+4.1%	*CARBAPENEMS*	8.8%	11.0%	+2.2%
meropenem	10.6%	21%	+10.4%	*meropenem*	11.0%	15.2%	+4.2%	*meropenem*	7.5%	10.5%	+3.0%
ertapenem	0.7%	0%	-0.7%	*ertapenem*	0.2%	0.8%	+0.6%	*imipenem/cilastatin*	1.3%	0.5%	-0.8%
imipenem/cilastatin	2.5%	0%	-2.5%	*imipenem/cilastatin*	1.0%	0.3%	-0.7%				
CEPHALOSPORINS	6.6%	11.0%	+4.4	*CEPHALOSPORINS*	19.8%	21.5%	+1.7%	*CEPHALOSPORINS*	19.7%	21.5%	+1.8%
cefazolin	1.7%	2.2%	+0.5%	*cefazolin*	0%	0.9%	+0.9%	*ceftriaxone*	4.2%	5.8%	+1.6%
ceftazidime+avibactam	0%	5.1%	+5.1%	*ceftolozane+tazobactam*	0%	0.1%	+0.1%	*cefepime*	7.1%	9.9%	+2.8%
ceftriaxone	2.7%	2.4%	-0.3%	*ceftritxone*	17.4%	19.8%	+2.4%	*ceftazidime*	5.6%	4.6%	-1.0%
ceftazidime	0.9%	0.1%	-0.8%	*cefepime*	0.9%	0.5%	-0.4%	*cefotaxime*	2.8%	1.0%	-1.8%
ceftolozane+tazobactam	1.3%	1.2%	-0.1%	*ceftazidime*	1.5%	0.0%	-1.5%	*ceftobiprole*	0%	0.12%	+0.12%
				ceftazidime+avibactam	0%	0.2%	+0.2%	*cefaroline fosamil*	0%	0.1%	+0.1%
MACROLIDES	0.9%	0.7%	-0.2%	*MACROLIDES*	3.8%	7.4%	+3.6%	*MACROLIDES*	11.7%	17.4%	+5.7%
clarithromycin	0.8%	0.6%	-0.2%	*clarithromycin*	3.6%	6.8%	+3.2%	*clarithromycin*	7.2%	14.0%	+6.8%
azithromycin	0.1%	0.1%	0%	*azithromycin*	0.2%	0.6%	+0.4%	*azithromycin*	4.5%	3.4%	-1.1%
POLYMYXINS	27.6%	26.7%	-0.9%	*POLYMYXINS*	1.3%	4.6%	+3.3%	*POLYMYXINS*	4.4%	2.0%	-2.4%
colistin				*colistin*				*colistin*			
LINCOSAMIDES	0%	1.0%	+1.0%	*LINCOSAMIDES*	0.4%	0%	-0.4%	*LINCOSAMIDES*	0.6%	0.9%	+0.3%
clindamycin				*clindamycin*				*clindamycin*			

Table S4. Annual consumption, expressed in % of total DDD antibiotics consumed, of fluoroquinolones, carbapenems, cephalosporins, macrolides, polymyxins, and lincosamides in some hospital wards of U.H. Ruggi in 2017 versus 2019.

HOSPITAL WARD: ***OBSTETRICS and GYNAECOLOGY***	***% of total DDD antibiotics consumed 2017***	***% of total DDD antibiotics consumed 2019***	***Variation % in 2019 vs. 2017***	*HOSPITAL WARD:* ***UROLOGY***	***% of total DDD antibiotics consumed 2017***	***% of total DDD antibiotics consumed 2019***	***Variation % in 2019 vs. 2017***	*HOSPITAL WARD:* ***INTENSIVE CARDIAC CARE UNIT***	***% of total DDD antibiotics consumed 2017***	***% of total DDD antibiotics consumed 2019***	***Variation % in 2019 vs. 2017***
FLUOROQUINOLONES<br>*levofloxacin*<br>*ciprofloxacin*	*5.1%*<br>*0.7%*<br>*4.4%*	*4.4%*<br>*0.4%*<br>*4.0%*	*-0.7%*<br>*-0.3%*<br>*-0.4%*	*FLUOROQUINOLONES*<br>*levofloxacin*<br>*ciprofloxacin*	*13.0%*<br>*3.9%*<br>*9.1%*	*15.2%*<br>*3.1%*<br>*12.1%*	*+2.2%*<br>*-0.8%*<br>*+3.0%*	*FLUOROQUINOLONES*<br>*levofloxacin*<br>*ciprofloxacin*	*16.9%*<br>*8.3%*<br>*8.6%*	*11.3%*<br>*7.5%*<br>*3.8%*	*-5.6%*<br>*-0.8%*<br>*-4.8%*
CARBAPENEMS<br>*meropenem*	*3.1%*	*2.4%*	*-0.7%*	*CARBAPENEMS*<br>*meropenem*<br>*imipenem/cilastatin*	*10.4%*<br>*7.3%*<br>*3.1%*	*8.7%*<br>*8.7%*<br>*0%*	*-1.7%*<br>*+1.4%*<br>*-3.1%*	*CARBAPENEMS*<br>*meropenem*	*5.6%*<br>*5.6%*	*11.9%*<br>*11.9%*	*+6.3%*<br>*+6.3%*
CEPHALOSPORINS<br>*cefazolin*<br>*ceftriaxone*<br>*cefotaxime*<br>*cefaclor*	*16.7%*<br>*0%*<br>*16.4%*<br>*0.2%*<br>*0.1%*	*45.5%*<br>*24%*<br>*21.4%*<br>*0.1%*<br>*0%*	*+28.8%*<br>*+24%*<br>*+5.0%*<br>*-0.1%*<br>*-0.1%*	*CEPHALOSPORINS*<br>*cefepime*<br>*ceftriaxone*<br>*cefotaxime*	*58.1%*<br>*0%*<br>*58.1%*<br>*0%*	*51.4%*<br>*0.2%*<br>*50.6%*<br>*0.6%*	*-6.7%*<br>*+0.2 %*<br>*-7.5%*<br>*+0.6%*	*CEPHALOSPORINS*<br>*cefazolin*<br>*ceftriaxone*<br>*ceftazidime*<br>*ceftaroline fosamil*	*29.6%*<br>*24.4%*<br>*5.0%*<br>*0.2%*<br>*0%*	*50.4%*<br>*40.7%*<br>*8.7%*<br>*0.7%*<br>*0.3%*	*+20.8%*<br>*+16.3%*<br>*+3.7%*<br>*+0.5%*<br>*+0.3%*
MACROLIDES<br>*clarithromycin*<br>*azithromycin*<br>*josamycin*	*6.4%*<br>*6.4%*<br>*0%*<br>*0%*	*6.2%*<br>*4.2%*<br>*0.3%*<br>*1.7%*	*-0.2%*<br>*-2.2%*<br>*+0.3%*<br>*+1.7%*	*MACROLIDES*<br>*clarithromycin*<br>*azithromycin*	*0.9%*<br>*0.8%*<br>*0.1%*	*5.2%*<br>*5.2%*<br>*0%*	*+4.3%*<br>*+4.4%*<br>*-0.1%*	*MACROLIDES*<br>*clarithromycin*	*1.4%*<br>*1.4%*	*1.8%*<br>*1.8%*	*+0.4%*<br>*+0.4%*
POLYMYXINS<br>*colistin*	*0%*	*0%*	_____	*POLYMYXINS*<br>*colistin*	*0%*	*0%*	------	*POLYMYXINS*<br>*colistin*	*2.0%*	*4.1%*	*+2.1%*
LINCOSAMIDES<br>*clindamycin*	*0%*	*0.1%*	*+0.1%*	*LINCOSAMIDES*<br>*clindamycin*	*1.3%*	*0%*	*-1.3%*	*LINCOSAMIDES*<br>*clindamycin*	*0%*	*0.7%*	*+0.7%*

Table S5. Annual consumption, expressed in % of total DDD antibiotics consumed, of fluoroquinolones, carbapenems, cephalosporins, macrolides, polymyxins, and lincosamides in some hospital wards of U.H. Ruggi in 2017 versus 2019.

HOSPITAL WARD: EMERGENCY SURGERY	*% of total DDD antibiotics consumed 2017*	*% of total DDD antibiotics consumed 2019*	*Variation % in 2019 vs. 2017*	*HOSPITAL WARD: GENERAL SURGERY*	*% of total DDD antibiotics consumed 2017*	*% of total DDD antibiotics consumed 2019*	*Variation % in 2019 vs. 2017*	*HOSPITAL WARD: EMERGENCY MEDICINE*	*% of total DDD antibiotics consumed 2017*	*% of total DDD antibiotics consumed 2019*	*Variation % in 2019 vs. 2017*
FLUOROQUINOLONES	*16.3%*	*10.2%*	*-6.1%*	*FLUOROQUINOLONES*	*10.3%*	*4.2%*	*-6.1%*	*FLUOROQUINOLONES*	*14.0%*	*6.7%*	*-7.3%*
levofloxacin	*7.2%*	*3.6%*	*-3.6%*	*levofloxacin*	*2.7%*	*1.7%*	*-1.0%*	*levofloxacin*	*6.3%*	*4.3%*	*-2.0%*
ciprofloxacin	*9.1%*	*6.6%*	*-2.5%*	*ciprofloxacin*	*7.6%*	*2.5%*	*-5.1%*	*ciprofloxacin*	*7.7%*	*2.4%*	*-5.3%*
CARBAPENEMS	*14.1%*	*9.6%*	*-4.5%*	*CARBAPENEMS*	*10.7%*	*3.5%*	*-7.2%*	*CARBAPENEMS*	*18.7%*	*20.1%*	*+1.4%*
meropenem	*10.4%*	*9.2%*	*-1.2%*	*meropenem*	*4.9%*	*3.2%*	*-1.7%*	*meropenem*	*15.4%*	*20.0%*	*+4.6%*
ertapenem	*2.9%*	*0.4%*	*-2.5%*	*ertapenem*	*5.7%*	*0.3%*	*-5.4%*	*ertapenem*	*0.1%*	*0.04%*	*-0.06%*
imipenem/cilastatin	*0.8%*	*0%*	*-0.8%*	*imipenem/cilastatin*	*0.1%*	*0%*	*-0.1%*	*imipenem/cilastatin*	*3.2%*	*0%*	*-3.2%*
CEPHALOSPORINS	*16.3%*	*18.6%*	*+2.3%*	*CEPHALOSPORINS*	*37.0%*	*50.7%*	*+13.7%*	*CEPHALOSPORINS*	*28.3%*	*26.2%*	*-2.1%*
cefazolin	*0.16%*	*0.5%*	*+0.34%*	*cefazolin*	*26.2%*	*33.0%*	*+6.8%*	*cefazolin*	*0%*	*0.04%*	*+0.04%*
ceftriaxone	*15.4%*	*14.0%*	*-1.4%*	*ceftriaxone*	*10.0%*	*12.4%*	*+2.4%*	*ceftriaxone*	*26.4%*	*24.4%*	*-2.0%*
cefepime	*0.08%*	*0%*	*-0.08%*	*cefepime*	*0.4%*	*1.7%*	*+1.3%*	*cefepime*	*0.1%*	*0.5%*	*+0.4%*
ceftazidime	*0.66%*	*2.9%*	*+2.24%*	*ceftazidime*	*0.4%*	*3.6%*	*+3.2%*	*ceftazidime*	*1.8%*	*0.9%*	*-0.9%*
ceftazidime+avibactam	*0%*	*1.1%*	*+1.1%*					*ceftazidime/avibactam*	*0%*	*0.3%*	*+0.3%*
								ceftaroline fosamil	*0%*	*0.06%*	*+0.06%*
MACROLIDES	*0.6%*	*0.8%*	*+0.2%*	*MACROLIDES*	*0.4%*	*1.2%*	*+0.8%*	*MACROLIDES*	*5.6%*	*10.9%*	*+5.3%*
clarithromycin	*0.57%*	*0.79%*	*+0.22%*	*clarithromycin*	*0.2%*	*1.0%*	*+0.8%*	*clarithromycin*	*5.3%*	*10.0%*	*+4.7%*
azithromycin	*0.03%*	*0.01%*	*-0.02%*	*azithromycin*	*0.2%*	*0.2%*	*0%*	*azithromycin*	*0.3%*	*0.9%*	*+0.6%*
POLYMYXINS	*1.9%*	*2.6%*	*+0.7%*	*POLYMYXINS*	*1.5%*	*1.5%*	*0%*	*POLYMYXINS*	*1.8%*	*0.9%*	*-0.9%*
colistin				*colistin*				*colistin*			
LINCOSAMIDES	*2.0%*	*0.7%*	*-1.3%*	*LINCOSAMIDES*	*0.7%*	*0.4%*	*-0.3%*	*LINCOSAMIDES*	*0.5%*	*0.4%*	*-0.1%*
clindamycin				*clindamycin*				*clindamycin*			
